# Evaluation of anxiety in a group of adult patients attending a dental surgery unit

**DOI:** 10.1186/s12903-025-05606-1

**Published:** 2025-03-07

**Authors:** Mayra Schemel-Suarez, Sonia Egido-Moreno, Isabel Martínez-Lizan, José López-López

**Affiliations:** 1 Departament of Odontostomatology, Faculty of Medicine and Health Sciences (Dentistry), University of Barcelona, Barcelona, Spain; 2 Departament of Odontostomatology, Faculty of Medicine and Health Sciences (Dentistry), University of Barcelona, C/Feixa Llarga s/n, Pavelló de Govern, 2a planta L’Hospitalet de Llobregat, Barcelona, 08907 España

**Keywords:** Blood pressure, Dental anxiety, Fear, Heart rate, Pain perception, Prevalence, Tooth extraction

## Abstract

**Background:**

Dental visits can cause anxiety and fear in some patients. Dental anxiety is considered a state of apprehension tied to a sensation of a loss of control and linked to a feeling that something “terrible” might happen during the dental treatment, this generates discomfort in the patient promoting that some of them avoid the dental visit, which in turns delays seeking treatment thus worsening the prognosis of oral diseases, for this reason the objective of this study is to determine the prevalence of anxiety among individuals attending an initial consultation at a dental surgery unit.

**Methods:**

The selection of patients was performed during the initial consultation carried out in the Medicine, Surgery and Oral Implantology unit and the following data was registered: hemodynamic parameters (blood pressure, heart rate and oxygen saturation), weight, pain associated to the patient’s reason for consultation, past dental experience, also State-Trait Anxiety Inventory/State-scale (STAI-S) and the modified dental anxiety scale (MDAS) questionnaire was applied to determine the level of anxiety. A descriptive analysis, Mann Whitney test, one way ANOVA, and two-way ANOVA were executed for the statistical analysis.

**Results:**

The sample consisted in 143 patients, 81 women and 62 men, the average age was 27.37 years old, a statistically significant difference was found in the average scores of anxiety between the group of men and women (*p* < 0.05); being the average of anxiety (STAI-S and MDAS) higher in women. A statistically significant difference was found between the values of STAI-S and the pain characteristic, finding that anxiety was higher when the patient had pain at the time of consultation (*p* = 0.001), although this was not found with the values of MDAS.

**Conclusion:**

The results of this study show that dental anxiety remains a significant issue for almost one third of the sample studied, anxiety is higher in women and may not be linked to previous negative dental experiences.

## Background

Dental visits can generate anxiety and fear in some patients. Dental anxiety is considered a state of apprehension tied to a sensation of loss of control that is linked to feelings that something terrible might happen during the dental treatment, conversely, dental fear is considered a normal emotional reaction to one or more specific threatening stimuli related to the dental practice [1]. A recent meta-analysis estimates the global prevalence of dental fear in adults to be 15.3% for any level of fear and 12.4% for high levels of dental fear [2]. The prevalence of dental anxiety is between 4 and 42.1% [3–7]. Sometimes the epidemiologic studies where the prevalence of dental anxiety is evaluated might use the terms anxiety and dental fear in an interchangeable manner [1], therefore obtaining the exact prevalence of each one is difficult [1, 2, 7, 8]. Different instruments have been designed to evaluate a patient’s level of anxiety and fear, these vary from a simple question to scales more complex, the most commonly used instruments include the modified dental anxiety scale (MDAS), the dental anxiety scale (DAS), the dental fear scale (DFS), the state trait anxiety inventory (STAI), among others [1, 2, 7, 8]. This variability in instruments could also explain the differences in anxiety/fear prevalence observed across various studies [2].

The etiology of dental anxiety is multifactorial and among them are: negative experiences in previous dental treatments, socioeconomic factors, the attitude and previous anxiety of the patient’s parents, the character and personality of the patient such as shyness and/or tendencies to negative emotions and other psychological disorders [9].

Anxiety triggers the autonomic nervous system, leading to an increase in involuntary bodily functions such as blood pressure, heart rate, gastrointestinal hypermotility, and cardiac expenditure [10]. All these symptoms generate discomfort in patients, contributing to the avoidance of dental consultations for some individuals. Consequently, this avoidance may delay seeking dental treatment, worsening the prognosis of oral diseases, and creating a vicious cycle [11]. Not obtaining dental treatment at the appropriate time may lead to an increase in disease severity, requiring more complex treatment, and consequently heightening the patient’s anxiety [12]. Additionally, a patient with dental anxiety that is not properly managed may have other types of inconveniences such as a decline in the threshold of pain, an increase in treatment complications, decrease in the time of recuperation and a heightening in post operatory pain [10].

Dental anxiety not only concerns patients but also generates challenges on dentist causing a decrease in patient’s collaboration, increase in treatment’s time and the potential to make an incorrect diagnosis and/or treatment for instance an erroneous pulp vitality assessment or mistakes in treatment planning [11].

Given the importance of assessing levels of dental anxiety in patients, the objective of this study is to determine the prevalence of anxiety among individuals attending an initial consultation at a dental surgery unit.

## Methods

### Study sample

A descriptive cross-sectional study was carried out in the Medicine, Surgery and Oral Implantology unit at the Dental Hospital University of Barcelona (Hospital Odontològic Universitat de Barcelona), during January 2022 to July 2023, with the previous approval of the Hospital’s ethical committee (CEIm HOUB- approval number 18/2018, Acta 26-6-2018) and according with principles established in the Helsinki declaration. The sample size was calculated to determine the prevalence of dental anxiety in patients attending an initial consultation. Assuming an estimated anxiety prevalence of 10% [13], a 95% confidence level, and a 0.05 precision, the sample size was determined using the formula for proportions: n = Z^2^⋅p⋅(1 − p)/d^2^ [14]. The minimum required sample size was calculated to be 138 participants.

Inclusion criteria: Individuals 18 years old or older, from both genders that wished to voluntarily participate in the study and that required the extraction of third molars.

Exclusion criteria: Individuals with systemic diseases classified as ASA III or IV that contraindicate the procedure, pregnant women, individuals who did not understand or could not respond to the questionnaire, patients needing extraction of a tooth other than a third molar or more than two third molars simultaneously, and those who have had a third molar extraction within the past twelve months.

### Study design

The process of patient selection was performed during the initial consultations in the Medicine, Surgery and Oral Implantology unit applying the inclusion and exclusion criteria. The individuals who met the general requirements were carefully informed about the study’s details and procedure; they were also given an information sheet about the study. The patients who agreed to participate in the investigation signed an informed consent. The same day, once the patient`s anamnesis was completed, hemodynamic parameters were taken (blood pressure, heart rate and oxygen saturation) using a vital signs monitor (Welch Allyn Spot Vital Signs LXi, Welch Allyn, NY, USA), the patient’s weight was registered, previous dental experience was determined with a questionnaire and assessment of pain associated with the reason for consultation was recorded using a visual analogue scale (VAS).

During oral exploration the Simplified Oral Higiene Index (OHI-S) and the decayed, missing and filled teeth index (DMFT) were evaluated and registered. Additionally, a STAI-S and MDAS questionnaires were provided to determine the level of anxiety. In this initial consultation, only anamnesis and examination were conducted; no other treatments were administered.

### Questionnaires

#### The state-trait anxiety inventory (STAI)

The State-Trait Anxiety Inventory [15] is used to measure through an auto evaluation the presence and severity of the actual symptoms of anxiety and the generalized tendency to be anxious, the inventory is composed of two scales: a state and a trait questionnaire, the Anxiety State scale (S-Anxiety) assesses the current state of anxiety, asking how the evaluated individual feels “in this moment”, using items that measure subjective feelings of apprehension, tension, nervousness, worry and activation/excitement of the autonomic nervous system [16].

The Anxiety Trait scale (T-Anxiety) evaluates relatively stable aspects of “anxiety tendency” that includes general states of calmness, confidence and security. Each inventory consists of 20 questions with 4 possible answers and a score from 0 (nothing) to 3 (a lot) and the total scores can go from 20 to 80 points [15], the present investigation only used the Anxiety State scale. A cut point value of 39 to 40 points has been suggested to detect clinically significant symptoms in the Anxiety State scale; however, other studies have suggested a higher cut point value, of 54 to 55 for older adults. The Spanish version of the STAI has been validated, achieving a Cronbach’s alpha reliability of 0.90 [17].

#### The modified dental anxiety scale (MDAS)

The modified dental anxiety scale (MDAS) is an auto evaluation questionnaire about dental anxiety that contains 5 items, each one with 5 possible answers that reflects in order an increase of anxiety (no anxiety to extremely anxious), the total score can go from 5 points to a maximum of 25 points. The lower limit to define an individual with extreme anxiety is 19 points and to define the presence of anxiety the score must be higher than 10 points [2, 18]. The internal reliability of the spanish MDAS ranges between 0.80 and 0.85 [19].

#### Visual analogous scale (VAS)

A visual analogous scale was used where 0 represents no presence of pain and 10 the presence of the worst imaginable pain [20], the patient marked on the line the level of pain perceived at the time of consultation. The marked position on the line was measured in mm and a value was assigned that ranged from 0 mm to 10 mm.

#### Previous dental experience

The following question was asked: “What would you say has been your previous experience at the dentist?” with three possible answers Good, Average or Bad. The patient classified the experience as “Good” when the reason for consultation was resolved satisfactorily and no complication or unpleasantness was perceived during treatment/evaluation; “Average” when the treatment was performed but certain inconvenience was felt due to the unpleasant sensation perceived during treatment/evaluation; and “Bad” when the patient experienced significant pain during treatment/evaluation or when the reason for consultation remained unresolved.

### Statistical analysis

The results are presented as frequencies, percentages, mean, and standard deviation. Differences in means between groups were assessed using one-way ANOVA. In cases where the data did not follow a normal distribution, the Mann-Whitney test was employed. Interaction between variables was analyzed using a two-way ANOVA. A significance level of α = 5% was applied to all analyses. The Statistical Package for Social Sciences (SPSS) version 28.0 (IBM corporation, Armonk, NY, USA) was used for data analyses.

## Results

**Fig. 1:**
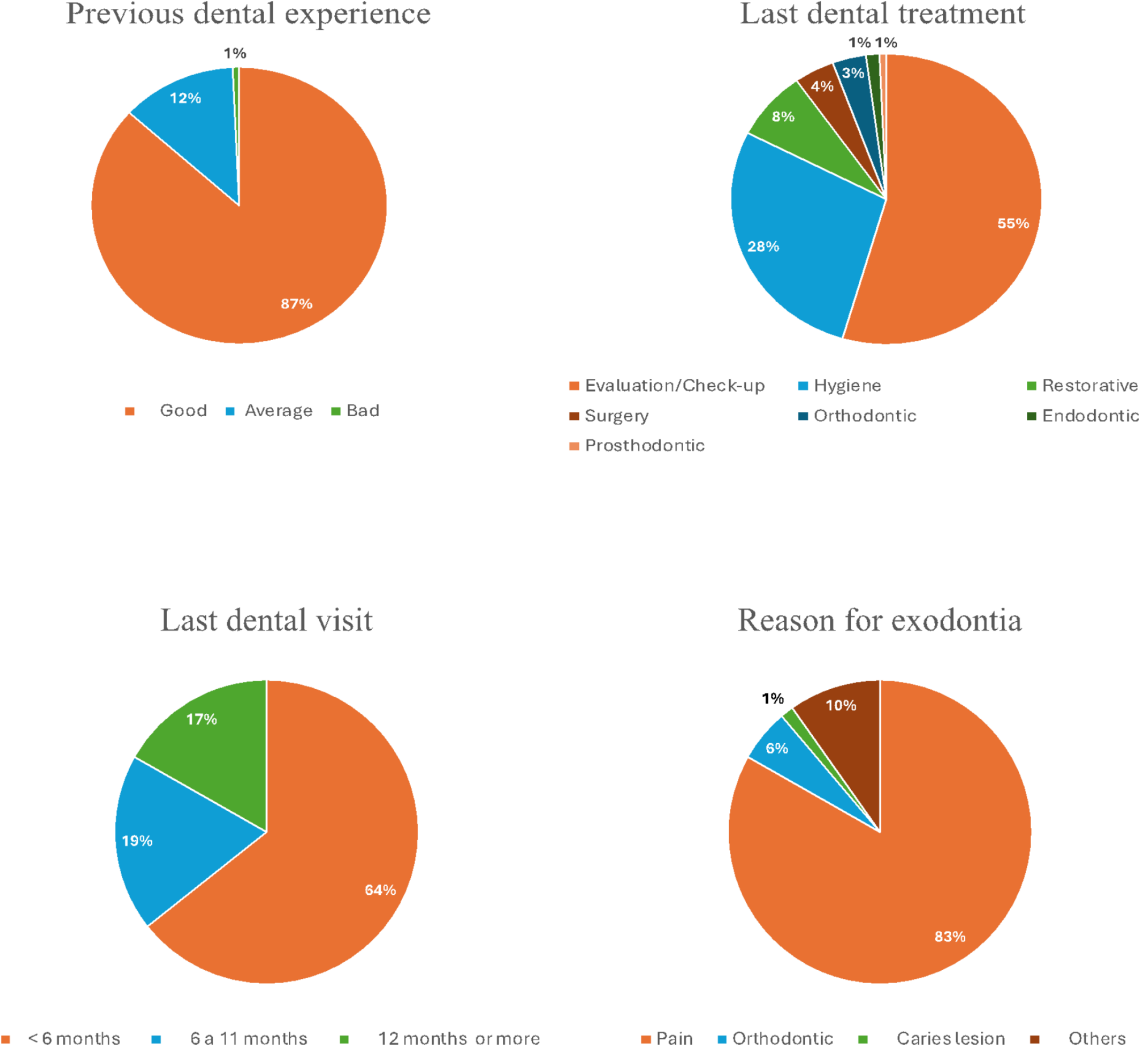
Sample characteristics regarding the dental consultation

The sample consisted of 143 patients, 81 (56.64%) women and 62 (43.36%) men, no statistical difference was found between the proportion of men and women (*p* = 0.112), the mean age was 27.37 years old, 124 (86.71%) individuals referred that their previous experience at the dentist was “good” and 92 (64.34%) patients of the total sample went to the dentist less than 6 months previous to the current consultation, the most common type of last treatment received by 78 (54.55%) patients was an evaluation or a regular check-up, the characteristics of the sample are shown in Table 1; Fig. 1.

The mean score of anxiety STAI-S of the sample was 35.07, 43 (30%) of the patients had a score greater or equal to 39 which is indicative of anxiety. The mean MDAS score was 10.95, with scores between 10 and 18 points (mild anxiety) in 80 (55.94%) of the individuals and scores greater or equal to 19 points (extreme anxiety) in 7 (4.90%) individuals.

The outcome of anxiety STAI-S for women was 36.7 and 32.9 points for men, finding a statistically significant difference between the groups (*p* = 0.006; Z=-2.742); the level of anxiety (STAI-S) being higher in women. The average score of anxiety MDAS in women was 11.8 and in men was 9.7, a statistically significant difference was found between the groups (*p* < 0.001, Z=-3.370), women had a higher level of anxiety (MDAS) than men (Table 2). The age variable was distributed in ranges of 10 years, as follows: 18 to 27 years old, 28 to 37 years old, 38 to 47 years old and 48 years and older (Table 2), no significant difference was found when analyzing the variables age and values of STAI-S and MDAS (*p* = 0.48, F = 0.825; *p* = 0.536; F = 0.729 respectively). When evaluating the variable anxiety and level of education no significant differences were found for STAI-S (*p* = 0.858; F = 0.255) and for MDAS (*p* = 0.655; F = 0.541), values can be found in Table 2. The results of oral hygiene and anxiety were evaluated (Table 2) and no significant differences were found between them; considering that only one individual had a poor hygiene score, the analysis was solely performed with the categories good and fair STAI-S (*p* = 0.519; Z=-0.644) and MDAS (*p* = 0.256; Z=-1.136). When conducting the factorial analysis of STAI-S, MDAS, gender, and OHI-S categories (as shown in Table 3), no significant interactions were observed among them as indicated by the global results (STAI-S *p* = 0.061; MDAS *p* = 0.220), however, when performing multiple pairwise comparison a significant difference was found between a higher STAI-S score in women and a OHI-S good classification (*p* = 0.001), also a significant difference was found between a higher MDAS score in women and an OHI-S good classification (*p* = 0.003) and an OHI-S fair classification (*p* = 0.026).

Heart rate was classified as normal and high (Table 2), no difference was found between the anxiety scores and the heart rate classification STAI-S (*p* = 0.361; Z= -0.914) and MDAS (*p* = 0.168; Z= -1.379), also not significant difference was found between the anxiety values and the classification of high-normal blood pressure and optimal-normal blood pressure (STAI *p* = 0.270 Z= -1.103; MDAS *p* = 0.08 Z= -1.750) Table 2; the classification used is based on the most recent international guidelines for hypertension (high-normal blood pressure: systolic ≥130mmHg and or diastolic ≥85mmHg) [21, 22].

A significant difference was found between the values of STAI-S and the pain variable, being anxiety higher when the patient had pain at the time of consultation (*p* = 0.001; Z=-3.248), although this was not found with MDAS values (*p* = 0.144; Z=-1.460) Table 2. However, upon conducting multiple pairwise comparisons involving the variables gender and pain (Table 3), a statistically significant difference was observed. Specifically, there was a significant difference between anxiety levels (STAI-S and MDAS) and the absence of pain in women, indicating that anxiety is significantly higher in women without pain (STAI-S *p* = 0.012; MDAS *p* = 0.008). It was also analyzed if there was any interaction between the variables anxiety, time passed since the last dental consultation and treatment received at the last dental visit and no statically significant interaction was found (Table 4).

**Table 1:** Characteristics of the sample

	Gender		Age				Total
	Women	Men	18–27	28–37	38–47	> 48	
**n**	81	62	88	40	10	5	143
**Age**	27.00 ± 8.74	27.85 ± 9.58	NA	NA	NA	NA	27.37 ± 9.09
**Weight (Kg)**	61.01 ± 12.08	87.74 ± 115.06	75.38 ± 97.79	67.40 ± 13.69	72.40 ± 16.16	65.80 ± 13.97	66.47 ± 13.37
**Systolic Blood pressure (mmHg)**	114 ± 9	123 ± 13	118 ±11	116 ± 12	117 ± 8	126 ± 25	118 ± 12
**Diastolic Blood pressure (mmHg)**	75 ± 6	78 ± 8	76 ± 8	75 ± 6	78 ± 5	80 ± 7	76 ± 8
**Heart rate (bpm)**	79 ± 11	72 ± 13	76 ± 13	76 ± 14	74 ± 9	83 ± 11	76 ± 13
**Oxygen Saturation (%)**	98 ± 2	98 ± 2	98 ± 2	98 ± 2	98 ± 1	97 ± 2	98
**OHI-S**	0.65 ± 0.78	0.76 ± 0.84	0.63 ± 0.80	0.78 ± 0.80	0.63 ± 0.72	1.19 ± 0.98	0.71 ± 0.82
**DMFT**	2	2	2	3	2	5	1
**VAS**	1.31 ± 2.33	1.24 ± 2.47	1.35 ±2.42	1.25 ± 2.56	0.75 ± 1.47	1.38 ± 2.34	1.27 ±2.39
High Blood Pressure*	7 [8.64%]	19 [30.64%]	17 [19.3%]	5 [12.5%]	2 [20%]	2 [40%]	26 [18.2%]
Previous Diagnosis of Hypertension*	1 [1.23%]	2 [3.22%]	1 [1.13%]	0 [0%]	0 [0%]	2 [40%]	3 [2.09%]

*Data presented as frequency and percentage

**Table 2:** Distribution of STAI-S and MDAS results by gender, age, level of education, OHI-S category, heart rate, blood pressure and pain

	*n*	%	STAI-S	*P* value	MDAS	*P* value
Gender*						
Women	81	56.64	36.7 (20–58)	**0.006**	11.8 (5–22)	**< 0.001**
Men	62	43.36	32.9 (20–62)		9.7 (5–23)	
Age**						
18–27	88	61.54	35.94 ± 9.62	0.48	10.60 ± 3.84	0.536
28–37	40	27.97	34.00 ± 9.08		11.70 ± 3.85	
38–47	10	6.99	33.70 ± 8.28		11.00 ± 3.80	
> 48	5	3.50	31.00 ± 6.28		11.00 ± 5.33	
Level of education**						
Primary	5	3.50	35.40 ± 6.66	0.858	11.60 ± 5.22	0.655
Secundary	79	55.24	35.63 ± 9.96		10.58 ± 3.73	
Universitary	37	25.88	34.05 ± 7.17		11.46 ± 3.43	
Vocational education and training	22	15.38	34.68 ± 10.06		11.27 ± 4.84	
OHI-S*						
Good (0–1.2)	107	74.82	35 (20–62)	0.519	10 (5–22)	0.256
Fair (1.3–3.0)	35	24.48	33 (20–51)		11 (5–22)	
Poor (3.1–6.0)	1	0.70	54 (54)		23 (23)	
Heart rate (bpm)*						
Normal (≤80 bpm)	88	61.54	34 (20–58)	0.61	10 (5–23)	0.168
High (> 80 ppm)	55	38.46	36 (20–62)		11(5–22)	
Blood Pressure (mmHg)*						
Optimal-Normal ≤129/84	117	81.82	35.0 (20–58)	0.270	9.0 (5–20)	0.08
High-Normal ≥130/85	26	18.18	31.5 (20–62)		11.0(5–23)	
Pain*						
Absence (0 EVA)	99	69.23	33.5 (20–57)	**0.001**	10 (5–23)	0.144
Presence (> 0 EVA)	44	30.77	38.5 (20–62)		11 (5–22)	
**Total**	143	100	35.07 ± 9.29 (20–62)		10.95 ± 3.88 (5–23)	

*Data presented as median (range)

**Data presented as mean ± standard deviation

**Table 3:** STAI-S and MDAS distribution by OHI-S category- gender and pain- gender

	Gender	*n*	STAI-S	*p* value*	MDAS	*p* value*
**OHI-S Category**						
Good	Women	61	37.74 ± 9.18	**0.001**	11.56 ± 3.63	**0.003**
	Men	46	31.93 ± 9.19		9.46 ± 3.29	
Fair	Women	20	33.65 ± 8.32	0.789	12.70 ± 4.52	**0.026**
	Men	15	34.47 ± 7.51		9.93 ± 2.98	
Total		142	34.94 ± 9.18		10.87 ± 3.76	
**Pain**						
Absence (0 VAS)	Women	54	35.57 ± 9.04	**0.012**	11.61 ± 3.66	**0.008**
	Men	45	31.02 ± 8.34		9.58 ± 3.75	
Presence (> 0 VAS)	Women	27	39.04 ± 8.94	0.675	12.30 ± 4.30	0.098
	Men	17	37.88 ± 9.59		10.35 ± 3.20	
Total	Total	143	35.07 ± 9.29		10.95± 3.88	

*Multiple pairwise comparison

**Table 4:** STAI-S and MDAS distribution by type of treatment received in the last dental visit and time passed since the last dental consultation

Type of treatment	Time passed since the last dental consultation	*n*	STAI-S	*p* value*	MDAS	*p* value*
Evaluation/Check-up	< 6 months	58	37.14 ±10.34	0.66	11.36± 4.52	0.233
	6 to 11 months	13	37.08± 8.17	0.270	12.00± 3.24	0.133
	≥ 12 months	7	32.57± 10.84	0.958	11.14± 2.96	0.882
Treatment**	< 6 months	34	33.44± 8.28	0.66	10.35± 3.51	0.233
	6 to 11 months	14	33.14± 10.48	0.270	9.71± 3.29	0.133
	≥ 12 months	17	32.35± 4.74	0.958	10.88± 3.44	0.882
Total		143	35.07± 9.29		10.95± 3.88	

*Multiple pairwise comparison

**Treatment (includes Hygiene, Restorative, Surgery, Orthodontic, Endodontic, Prosthodontic)

## Discussion

The results of the present study indicate that 30% of the sampled population exhibited anxiety, as measured by the STAI-S scale. Among them, 55.94% experienced mild anxiety, while only 4.9% reported extreme anxiety, as assessed by the MDAS scale. This finding is consistent with studies conducted in India [6] and the United States [3] where 4% and 7% of their sample had MDAS scores ≥ 19 points. It is important to note that 86% of the individuals in the current study reported that their past dental appointment was rated as “good”, furthermore, most of these individuals only underwent an evaluation or check-up during their last dental visit, therefore it can be interpreted that the anxiety showed at the time of the present study was not related to recent past negative dental experiences. Another finding of this investigation is the difference in anxiety scores between men and women, with women exhibiting higher levels of anxiety. This observation aligns with results from other studies [3, 7, 12, 23, 24]. In the literature, various theories explain the difference in anxiety disorder prevalence between men and women, typically categorized into psychosocial and biological factors; a recent systematic review [25] highlights that psychosocial factors, such as gender socialization, may partly influence psychosocial traits, making them risk factors for women and protective factors for men, with higher adherence to femininity linked to increased anxiety in women; however, it’s important to consider that research may be biased towards men, as many men adhering to gender norms may prefer self-sufficiency and avoid seeking help, potentially resulting in a lower reported proportion of men with anxiety in scientific studies [25]. Within the biological factors, the studies show that anxiety is determined by *genetic factors* (methylation of the SLC6A4 gen is higher in women when compared to men, which could be the base of a differential expression in the serotonin transporter SERT in women, this generates a greater prevalence of somatic disorders, since the serotonin pathway helps in mood regulation [26]), *hormonal factors* ( hormonal fluctuation in progesterone, estrogen and oxytocin [25]) and/or *neuroanatomic factors* (in women the central left amygdala is activated with stimuli and negative emotion in contrast on men is activated with positive emotion, which could have implications in emotional regulation [27]).

In the present investigation no significative difference was found between the variables anxiety and age, which is consistent with other studies [4, 12, 28]. However, there was a tendency to lower scores of anxiety as age increased. It is worth noting that the number of individuals above 37 years old in this study is lower due to the type of intervention that the participants sought. The average age of patients experiencing discomfort generated by the eruption of third molars and/or are interested in the extraction of third molars generally falls between 25 and 34 years old [29–31]. Similarly, no significant difference was found between the variables anxiety and oral hygiene, this could be attributed to the fact that the sample mainly comprised young patients with good oral hygiene, also 28% of the sample had a hygiene treatment as their last treatment resulting in minimal fluctuations in values. This finding does not align with the study by Hofer et al., which found a correlation between plaque-induced gingivitis and higher levels of anxiety. They attributed this to the patients’ ambivalence toward maintaining optimal oral hygiene, despite adhering to regular dental hygiene recall appointments [32]. It is important to mention that when a multiple analysis comparison was performed a statistically significant difference was found between a higher MDAS score, female gender, and a good and fair oral hygiene status, which points out once more the orientation of a higher anxiety level in women. No significant difference was found between groups with high heart rates, normal heart rates, and anxiety scores. The average heart rate was 76 bpm, which is lower than reported in other studies [33–35]. This may be because the patients only had a consultation and no other dental treatments on the same day, which probably minimized their stress and prevented activation of the autonomic nervous system. In contrast, other studies involved dental treatments on the same day as data collection, which could have influenced their results. When evaluating the variables blood pressure and anxiety, no significant differences was found between the patients with high blood pressure, optimal blood pressure and anxiety scores. It is important to highlight that the amount of patients with high-normal blood pressure is an 18.2% of the total sample and only 3% was previously diagnosed with high blood pressure. According to the International Society of Hypertension, 10 to 30% of individuals who visit a clinic for high blood pressure have white coat hypertension, while 10 to 15% have masked hypertension [21], this could coincide with the finding in the present investigation. Nonetheless, the European Society of Hypertension [22] in their latest hypertension management guide, recommend the performance of an opportunistic screening in all adults for the early detection of hypertension since many individuals are not aware of having this condition, furthermore to confirm hypertension they recommend doing the measurement in the clinic in at least 3 different visits and these data can be combined with house measurements to help determine if the changes in measurement are due to the white coat phenomenon, in the present study the patient was informed when they presented higher values of blood pressure and they were advised to attend their primary health care center for further assessment and following.

Statistically significant difference was found between a higher STAI-S score and the presence of pain, this can be related with the results obtained by Kankaanpää y col [36]., they observed that pain’s threshold and tolerance is lower in patients with dental anxiety or dental fear, they also concluded that women had lower tolerance to pressure pain and a lower pressure pain threshold compare to men when this was associated to dental anxiety or dental fear as well as fear of anticipation and treatment, whereas for men lower tolerance for pain was associated to dental anxiety or dental fear. Additionally, in the present study higher scores of anxiety were found in the women group with absence of pain, which can be explained by the general tendency of women to present higher anxiety scores as a whole.

The limitations of the present investigation are the sample size and the small representation of individuals in the 38 years and older age range, which can generate less information to analyze and correlate with anxiety scores, since previous dental experience and hygiene status tend to change as age increases. Additionally, only patients undergoing third molar extraction were included to ensure consistency in the type of treatment across the sample, standardizing patients’ expectations of the procedure. Third molar extraction is also the most common procedure in oral surgery and is frequently more complex, involving a flap, coronectomy, bone removal, or root separation. Given its complexity, it is often a new experience for patients, which may affect their anxiety levels.

## Conclusion

The results of the investigation reveal that one-third of the sample experienced anxiety according to the STAI-S scale, while half of the sample had mild anxiety based on MDAS scores. Anxiety levels were higher among women, but this was not associated with prior negative dental experiences. Additionally, patients with anxiety reported higher levels of pain, although their heart rates were not affected by anxiety scores.

## Data Availability

The datasets used and/or analysed during the current study are available from the corresponding author on reasonable request.

## Abbreviations

ASA: American Society of Anesthesiologists
ANOVA: Analysis of Variance
CEIm: Comité de Ética e Investigación con Medicamentos y Productos Sanitarios
DAS: Dental Anxiety Scale
DFS: Dental Fear Scale
DMFT: Decayed Missing Filled Tooth
HOUB: Hospital Odontologic Universitat de Barcelona
HR: Heart Rate
MDAS: Modified Dental Anxiety Scale
OHI-S: Simplified Oral Hygiene Index
SERT: Serotonin Transporter
SPSS: Statistical Package for Social Sciences
STAI: State-Trait Anxiety Inventory
VAS: Visual Analogous Scale

## References

[1] Grisolia BM, dos Santos APP, Dhyppolito IM, Buchanan H, Hill K, Oliveira BH. Prevalence of dental anxiety in children and adolescents globally: a systematic review with meta-analyses. Int J Paediatr Dent. 2021;31:168–83. 33245591 10.1111/ipd.12712

[2] Silveira ER, Cademartori MG, Schuch HS, Armfield JA, Demarco FF. Estimated prevalence of dental fear in adults: a systematic review and meta-analysis. J Dent. 2021;108 February. 10.1016/j.jdent.2021.103632 33711405

[3] White AM, Giblin L, Boyd LD. The prevalence of Dental anxiety in Dental Practice settings. J Dent Hyg. 2017;91:30–4. 29118148

[4] Nicolas E, Collado V, Faulks D, Bullier B, Hennequin M. A national cross-sectional survey of dental anxiety in the French adult population. BMC Oral Health. 2007;7:1–7. 17927808 10.1186/1472-6831-7-12 PMC2098754

[5] Enkling N, Marwinski G, Jöhren P. Dental anxiety in a representative sample of residents of a large German city. Clin Oral Investig. 2006;10:84–91. 16477408 10.1007/s00784-006-0035-6

[6] Thakur S, Kadam H, Jha S, Lall A, Kondreddy K, Singh A. Assessment of anxiety Associated with the Dental treatments on the quality of life: an Original Research. J Pharm Bioallied Sci. 2021;13(Suppl 2):S1713–6. 35018061 10.4103/jpbs.jpbs_108_21 PMC8686871

[7] Yakar B, Kaygusuz TÖ, Pırınçcı E. Evaluation of Dental anxiety and fear in patients who admitted to the Faculty of Dentistry: which patients are more risky in terms of Dental anxiety. Ethiop J Health Sci. 2019;29:719–26. 31741642 10.4314/ejhs.v29i6.8 PMC6842720

[8] Erten H, Akarslan ZZ, Bodrumlu E. Dental fear and anxiety levels of patients attending a dental clinic. Quintessence Int. 2006;37:304–10. 16594362

[9] Metin-gürsoy G, Haciomeroglu AB, Kale-varlık S. Evaluation of the relationship between anxiety levels and dental appearance. J Clin Pediatr Dentistry. 2023. 10.22514/jocpd.2023.018. 10.22514/jocpd.2023.018 37408345

[10] Hoffmann B, Erwood K, Ncomanzi S, Fischer V, O’Brien D, Lee A. Management strategies for adult patients with dental anxiety in the dental clinic: a systematic review. Aust Dent J. 2022;67:S3–13. 35735746 10.1111/adj.12926 PMC9796536

[11] Winkler CH, Bjelopavlovic M, Lehmann KM, Petrowski K, Irmscher L, Berth H. Impact of Dental anxiety on Dental Care Routine and oral-health-related quality of life in a German adult Population—A cross-sectional study. J Clin Med. 2023;12. 10.3390/jcm12165291 PMC10455740 37629334

[12] Saba Z, Katirci G. Relationship between dental anxiety levels and oral health among dental patients in Turkey: a cross-sectional study. BMC Oral Health. 2023;23:1–10. 37231452 10.1186/s12903-023-03041-8 PMC10214591

[13] Liddell A, Locker D. Gender and age differences in attitudes to dental pain and dental control. Community Dent Oral Epidemiol. 1997;25:314–8. 9332809 10.1111/j.1600-0528.1997.tb00945.x

[14] Pourhoseingholi MA, Vahedi M, Rahimzadeh M. Sample size calculation in medical studies. Gastroenterol Hepatol Bed Bench. 2013;6:14–7. 24834239 PMC4017493

[15] Spielberger CD, Gorsuch RL, Lushene R, Vagg PR, Jacobs GA. Manual for the state-trait anxiety inventory. Palo Alto, CA: Consulting Psychologists; 1983.

[16] Julian LJ. Measures of anxiety: state-trait anxiety inventory (STAI), Beck anxiety inventory (BAI), and hospital anxiety and depression scale-anxiety (HADS-A). Arthritis Care Res (Hoboken). 2011;63(Suppl 1 0 11):S467–72. 22588767 10.1002/acr.20561 PMC3879951

[17] Guillén-Riquelme A, Buela-Casal G. [Psychometric revision and differential item functioning in the state trait anxiety inventory (STAI)]. Psicothema. 2011;23:510–5. 21774907

[18] Humphris GM, Morrison T, Lindsay SJ. The Modified Dental anxiety scale: validation and United Kingdom norms. Community Dent Health. 1995;12:143–50. 7584581

[19] Coolidge T, Chambers MA, Garcia LJ, Heaton LJ, Coldwell SE. Psychometric properties of spanish-language adult dental fear measures. BMC Oral Health. 2008;8:1–8. 18474102 10.1186/1472-6831-8-15 PMC2391155

[20] Hayes MHS, Patterson DG. Experimental development of the graphic rating method. Psychol Bull. 1921;18:98–9.

[21] Unger T, Borghi C, Charchar F, Khan NA, Poulter NR, Prabhakaran D, et al. 2020 International Society of Hypertension Global Hypertension Practice Guidelines. Hypertension. 2020;75:1334–57. 32370572 10.1161/HYPERTENSIONAHA.120.15026

[22] Mancia G, Kreutz R, Brunström M, Burnier M, Grassi G, Januszewicz A, et al. 2023 ESH guidelines for the management of arterial hypertension the Task Force for the management of arterial hypertension of the European Society of Hypertension: endorsed by the International Society of Hypertension (ISH) and the European renal associat. J Hypertens. 2023;41:1874–2071. 37345492 10.1097/HJH.0000000000003480

[23] Candido MC, Andreatini R, Zielak JC, de Souza JF, Losso EM. Assessment of anxiety in patients who undergo surgical procedures for tooth implants: a prospective study. Oral Maxillofac Surg. 2015;19:253–8. 25572978 10.1007/s10006-014-0480-3

[24] Suleiman AR, Efunkoya AA, Omeje KU, Amole IO. The effect of dental anxiety on surgical time of mandibular third molar disimpaction. Niger J Clin Pract. 2021;24:1430–7. 34657006 10.4103/njcp.njcp_501_20

[25] Farhane-Medina NZ, Luque B, Tabernero C, Castillo-Mayén R. Factors associated with gender and sex differences in anxiety prevalence and comorbidity: a systematic review. Sci Prog. 2022;105:1–30. 10.1177/00368504221135469 PMC10450496 36373774

[26] Palma-Gudiel H, Peralta V, Deuschle M, Navarro V, Fañanás L. Epigenetics-by-sex interaction for somatization conferred by methylation at the promoter region of SLC6A4 gene. Prog Neuropsychopharmacol Biol Psychiatry. 2019;89:125–31. 30201454 10.1016/j.pnpbp.2018.09.002

[27] Gardener EKT, Carr AR, Macgregor A, Felmingham KL. Sex differences and emotion regulation: an event-related potential study. PLoS ONE. 2013;8:e73475. 24204562 10.1371/journal.pone.0073475 PMC3813629

[28] Alghareeb Z, Alhaji K, Alhaddad B, Gaffar B. Assessment of Dental anxiety and hemodynamic changes during different Dental procedures: a Report from Eastern Saudi Arabia. Eur J Dent. 2022;16:833–40. 34991162 10.1055/s-0041-1740222 PMC9683887

[29] Dudde F, Barbarewicz F, Henkel K. Distribution and impaction patterns of third molars in a sample of German population: retrospective analysis in a high turnover maxillofacial department. J Stomatol Oral Maxillofac Surg. 2024;125:101763. 38218335 10.1016/j.jormas.2024.101763

[30] Al-Madani SO, Jaber M, Prasad P, Maslamani MJM, Al. The patterns of impacted third molars and their Associated pathologies: a retrospective observational study of 704 patients. J Clin Med. 2024;13:1–17. 10.3390/jcm13020330 PMC10816724 38256464

[31] Sakakura H, Hayashi Y, Sugimoto K, Matsubara A. Relationship between age-related changes in mandibular third molar roots and the possibility of mental nerve paresthesia after tooth extraction. Int J Oral Maxillofac Surg. 2024;December:1–7. 10.1016/j.ijom.2023.12.008 38199951

[32] Hofer D, Thoma MV, Schmidlin PR, Attin T, Ehlert U, Nater UM. Pre-treatment anxiety in a dental hygiene recall population: a cross-sectional pilot study. BMC Oral Health. 2016;16:43. 27009086 10.1186/s12903-016-0198-8 PMC4806470

[33] Sorribes De Ramón LA, Ferrández Martínez AF, García Carricondo AR, Espín Gálvez F, Alarcón Rodríguez R. Effect of virtual reality and music therapy on anxiety and perioperative pain in surgical extraction of impacted third molars. J Am Dent Assoc. 2023;154:206–14. 36707274 10.1016/j.adaj.2022.11.008

[34] Turer OU, Ozcan M, Alkaya B, Demirbilek F, Alpay N, Daglioglu G, et al. The effect of mindfulness meditation on dental anxiety during implant surgery: a randomized controlled clinical trial. Sci Rep. 2023;13:1–11. 38066232 10.1038/s41598-023-49092-3 PMC10709419

[35] Yildirim O, Turkmenoglu K, Mollaoglu N. Assessment of relationship between hemodynamic changes and anxiety in patients during lower third molar surgery. J Coll Physicians Surg Pakistan. 2022;32:1524–8. 10.29271/jcpsp.2022.12.1524 36474368

[36] Kankaanpää R, Auvinen J, Rantavuori K, Jokelainen J, Karppinen J, Lahti S. Pressure pain sensitivity is associated with dental fear in adults in middle age: findings from the Northern Finland 1966 birth cohort study. Community Dent Oral Epidemiol. 2019;47:193–200. 30549076 10.1111/cdoe.12443

